# The association between the AST/ALT ratio and osteopenia or osteoporosis in patients with type 2 diabetes mellitus

**DOI:** 10.1530/EC-25-0086

**Published:** 2025-05-13

**Authors:** Yuan Zhang, Guanhua Chen, Shanshan Lv, Weimin Wang, Yali Jing

**Affiliations:** ^1^ Department of Endocrinology, Endocrine and Metabolic Disease Medical Center, Nanjing Drum Tower Hospital Clinical College of Nanjing University of Chinese Medicine, Nanjing, China; ^2^ Branch of National Clinical Research Centre for Metabolic Diseases, Nanjing, China; ^3^ Department of Endocrinology, Endocrine and Metabolic Disease Medical Center, Nanjing Drum Tower Hospital, Affiliated Hospital of Medical School, Nanjing University, Nanjing, China

**Keywords:** type 2 diabetes mellitus, AST/ALT ratio, osteopenia, osteoporosis

## Abstract

**Introduction:**

The aim of this study was to investigate the relationship between the AST/ALT ratio and osteopenia or osteoporosis in patients with type 2 diabetes mellitus (T2DM).

**Methods:**

A total of 589 patients with T2DM were divided into two groups based on T-score: T-score ≥ −1.0 group, normal bone mineral density and T-score < −1.0 group, osteopenia or osteoporosis (OP). The association between the AST/ALT ratio and osteopenia/OP was evaluated by multivariate analyses. The receiver operating characteristic (ROC) curves were used to estimate the diagnostic performance according to the area under the ROC curve (AUC).

**Results:**

The patients in the T-score < −1.0 group showed significantly higher AST/ALT level than those in the T-score ≥ −1.0 group (0.93 ± 0.16 vs 1.17 ± 0.24, *P* < 0.001). According to the interquartile range of the AST/ALT ratio, the participants were divided into four groups: Q1 (0.650, 0.874), Q2 (0.875, 0.999), Q3 (1.000, 1.173) and Q4 (1.174, 1.917). After adjustment for confounding factors, compared with Q1 of the AST/ALT level, subjects in Q3 and Q4 remained more likely to have osteopenia or osteoporosis (Q3, OR 3.478, 95% CI 1.641–7.411; Q4, OR 15.278, 95% CI 6.377–36.837). The AST/ALT ratio provided an AUC value of 0.81 (95% CI 0.77–0.84) for osteopenia or osteoporosis in patients with T2DM.

**Conclusion:**

An elevated AST/ALT ratio is associated with the evaluated risk of osteopenia/OP in patients with T2DM. The AST/ALT ratio, a practical and cost-effective biomarker, may be a potential predictor of osteopenia/OP in patients with T2DM.

## Introduction

Type 2 diabetes mellitus (T2DM) is a chronic metabolic disease that seriously affects human health and is a serious public health challenge with an increasing prevalence (1). Osteoporosis (OP) is a systemic bone disease characterized by low bone mass and damage to the microstructure of bone tissue, leading to increased bone fragility and susceptibility to fracture (2). The incidence of osteoporosis is in a period of rapid increase with the aging of the global population. Recently, accumulating studies have demonstrated that T2DM is associated with poor bone health, hyperglycemia, hyperlipidemia, vascular complications and glucose-lowering medications in patients with T2DM and could affect the normal bone metabolic homeostasis to varying degrees, for example, disrupting the balance of bone conversion mediated by osteoblasts and osteoclasts (3). Importantly, coexisting T2DM and OP could result in a higher risk of fracture, even death, posing a serious disease and socioeconomic burden (4, 5, 6). Therefore, it is critical to explore possible risk factors or biomarkers and intervene timely for osteopenia/osteoporosis in T2DM patients.

The metabolic dysfunction-associated steatotic liver disease (MASLD) has rapidly become the most common chronic liver disease globally (7). Previous studies have reported a significant relationship between the prevalence and severity of MASLD and endocrine disorders, and T2DM is an independent risk factor for the development of MASLD and the progression of liver fibrosis and cirrhosis (8). Bone remodeling is a continuous process between bone formation and bone resorption mediated by osteoblasts and osteoclasts, and any imbalance leads to metabolic bone disease. Increasing evidence suggests that MASLD decreases bone mineral density (BMD) and increases the risk of fragility fractures. For example, oxidative stress is an important mechanism of osteoporosis. Wang *et al.* reported that MAFLD itself may aggravate the inflammatory state in elderly OP people due to mitochondrial homeostasis imbalance, triggering attenuation of the body functions of OP patients (9). The possible mechanism of MASLD and bone health is a bidirectional flow of signals, such as endocrine and metabolic factors, between the liver and bone tissues, which work together to regulate metabolic functions in the liver and bone, suggesting a potential causal relationship between OP and MASLD (10, 11).

Metabolic diseases have always been thought to be a significant risk factor for osteoporosis, whereas fatty liver disease is usually associated with metabolic dysfunction and is a major predisposing factor in diabetes. Liver enzymes, especially alanine aminotransferase (ALT) and aspartate aminotransferase (AST), are essential indicators of liver function. The AST/ALT ratio, representing simultaneous changes in AST and ALT levels, is a widely adopted marker of liver fibrosis, and is also associated with obesity, hyperglycemia, insulin resistance and metabolic syndrome (12, 13, 14, 15). Moreover, an elevated AST/ALT ratio may be associated with systemic inflammation and oxidative stress, which can reflect not only liver function but also serve as an indicator of inflammatory status. Several studies have found the correlation between the AST/ALT ratio and muscle mass, demonstrating the AST/ALT ratio as a predictor of sarcopenia (16, 17, 18). A cross-sectional study in Koreans revealed a correlation between liver enzyme levels (including AST and ALT) and BMD of femoral neck (19). The AST/ALT ratio offers unique advantages by combining insights into systemic inflammation and liver function. Considering the strong association between bone and muscle (20), and the relatively high predictive performance of the AST/ALT ratio in metabolic diseases, we hypothesized that the AST/ALT ratio could likewise be a potential predictor of bone metabolic diseases.

To date, there are limited studies on the correlation between the AST/ALT ratio and osteopenia or osteoporosis in patients with T2DM. The aim of this study was to determine the association between the AST/ALT ratio and osteopenia/osteoporosis in T2DM patients.

## Materials and methods

### Study population

This study was a cross-sectional study. We retrospectively collected patients with T2DM, aged between 50 and 80 years, who were hospitalized in the Department of Endocrinology, Nanjing Drum Tower Hospital Clinical College of Nanjing University of Chinese Medicine between January 2022 and January 2024. All patients had been diagnosed with T2DM at baseline according to the 1999 World Health Organization (WHO) standards (21). We excluded patients with the following conditions: i) presence of type 1 diabetes or other types of diabetes; ii) acute complications of diabetes, such as diabetic hyperosmotic hyperglycemia syndrome, diabetic ketoacidosis and hypoglycemia; iii) patients with diseases which seriously affect bone metabolism and lead to secondary osteoporosis, such as past or present autoimmune diseases (rheumatoid arthritis), hematological diseases, hyper-/hypothyroidism, primary hyper-/hypoparathyroidism, rickets, cancer and Cushing syndrome; iv) medical treatment that could affect bone metabolism, including thiazolidinediones, calcium, calcitriol, diphosphonate, calcitonin, denosumab, teriparatide and glucocorticoid; v) disabled patients who cannot move or are bedridden for a long time; and vi) patients with incomplete data. The study followed the Declaration of Helsinki thoroughly and was approved by the medical research ethics committee of the Nanjing Drum Tower Hospital.

### Data collection

Information related to demographics, health status and function, blood biomarker measurements and BMD measurements were collected. We collected data related to demographics parameters (including age, gender, blood pressure and duration of diabetes). Body mass index (BMI) was calculated as weight divided by the square of height (kg/m^2^). In addition, the following data were obtained from the hospital information system: aspartate aminotransferase (AST), alanine aminotransferase (ALT), serum creatinine (Cr), blood urea nitrogen (BUN), hemoglobin A1c (HbA1c), fasting plasma glucose (FPG), postprandial 2h plasma glucose (2hPG), total cholesterol (TC), triglycerides (TG), serum 25-hydroxy vitamin D (25(OH)D) and serum osteocalcin (OC)*.*

### Bone mineral density measurement

BMD (g/cm^2^) was measured using dual energy X-ray absorptiometry (Lunar iDXA, USA). Measurements were made at three sites in each patient: femoral neck (FN), lumbar spine (LS) and left hip (LH). BMD was measured according to the World Health Organization criteria: normal, with a T-score ≥ −1.0; osteopenia, with a T-score between −1.0 and −2.5; and osteoporosis, with a T-score ≤ −2.5 (22). The final included participants were divided into two groups based on the T-score: T-score ≥ −1.0 group (normal BMD) and T-score < −1.0 group (osteopenia or osteoporosis).

### Statistical analysis

The baseline characteristics of involved participants were described by the mean (continuous variable) or proportion (categorical variable). Continuous variables with normal distribution were expressed as the mean ± standard deviation (SD), and independent samples *T*-test was used to compare the T-score ≥ −1.0 group with the T-score < −1.0 group. Continuous variables without a normal distribution were expressed as the median (interquartile range), and compared between the T-score ≥ −1.0 and T-score < −1.0 groups adopting the Kruskal–Wallis test. Categorical variables were shown as proportions and were compared using chi-squared tests. The AST/ALT level was divided into four quartiles and converted into conventional categorical variables, Q1 < 25%, Q2 25–50%, Q3 50–75% and Q4 ≥ 75%. The proportion of osteopenia/osteoporosis in patients with T2DM was examined by chi-square test to compare the above categorical variables. Spearman correlation analysis was used to analyze the correlation between the AST/ALT ratio and BMDs. After adjusting for potential confounders, the binary logistic regression analyses were performed to examine the correlation between the AST/ALT ratio quartiles and the risk of osteopenia or osteoporosis in all subjects and to estimate the ratio of odds ratios (ORs) and 95% confidence intervals (CIs). Receiver operating characteristic (ROC) curves were performed and area under the curve (AUC) values were estimated. All statistical analyses were performed using the SPSS 27.0. Statistics were deemed significant when *P* < 0.05.

## Results

**Figure 1:**
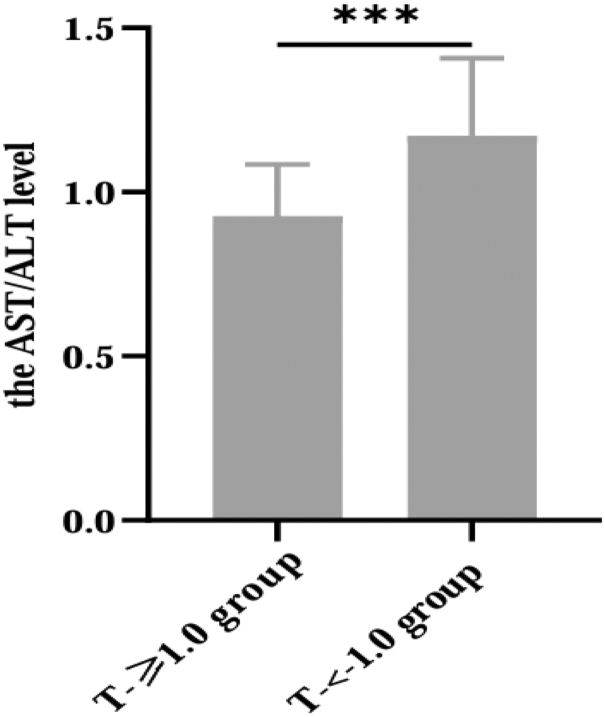
Difference in the AST/ALT ratio between the two groups. ***P < 0.001.

A total of 589 patients with T2DM were included in this study, including 227 women and 362 men, of which 310 had normal BMD (T-score ≥ −1.0) and 279 had osteopenia/osteoporosis (T-score < −1.0). The prevalence of T-score < −1 was higher in females than males among T2DM patients (74.0 vs 30.7%, *P* < 0.001). In the total population, compared with the T-score ≥ −1.0 group, age, duration of diabetes, SBP, OC, β-CTX and PINP levels of the T-score < −1.0 group were significantly higher (*P* < 0.05), while BMI, DBP, Cr, UA, 25(OH)D, femoral neck BMD, LH BMD and LS BMD were significantly lower (*P* < 0.05) (Table 1). Whereas, there was no significant difference in FPG, 2hPG, FCP, HbA1c, AST, BUN, TG and TC (Table 1). Notably, the level of the AST/ALT ratio was significantly higher in the T-score < −1 group (Fig. 1).

**Table 1:** Clinical characteristics of the study subjects.

Variables	T- ≥ −1.0 group (*n* = 310)	T- < −1.0 group (*n* = 279)	*P*-value
Age (year)	60.45 ± 6.18	67.58 ± 5.97	<0.001
Male (*n*, %)	251, 81.0%	111, 40.0%	<0.001
Smoking (*n*, %)	113, 36.5%	42, 15.1%	<0.001
Drinking (*n*, %)	70, 22.6%	26, 9.3%	<0.001
SBP (mmHg)	129.95 ± 16.76	133.76 ± 19.17	0.021
DBP (mmHg)	81.05 ± 9.74	76.67 ± 10.44	<0.001
BMI (kg/m^2^)	24.38 ± 2.18	23.27 ± 2.45	<0.001
Duration (year)	10.72 ± 5.93	13.33 ± 6.29	<0.001
FPG (mmol/L)	7.95 ± 2.30	7.82 ± 2.38	0.407
2hPG (mmol/L)	15.06 ± 4.00	15.54 ± 4.16	0.117
FCP (pmol/L)	572.57 ± 264.19	557.01 ± 313.14	0.202
HbA1c (%)	8.39 ± 2.00	8.35 ± 1.86	0.915
AST (U/L)	19.61 ± 7.32	19.63 ± 8.54	0.550
ALT (U/I)	21.91 ± 9.75	17.57 ± 9.27	<0.001
AST/ALT	0.93 ± 0.16	1.17 ± 0.24	<0.001
BUN (mmol/L)	6.10 ± 1.74	6.09 ± 1.68	0.973
Cr (umol/L)	63.68 ± 17.10	59.40 ± 15.98	<0.001
UA (umol/L)	338.37 ± 87.89	301.63 ± 79.30	<0.001
TG (mmol/L)	1.54 ± 1.17	1.36 ± 0.68	0.121
TC (mmol/L)	4.41 ± 1.12	4.46 ± 1.08	0.568
OC (ng/mL)	11.70 ± 3.73	14.15 ± 6.63	<0.001
β-CTX (ng/mL)	0.35 ± 0.17	0.43 ± 0.23	<0.001
PINP (ng/mL)	37.09 ± 14.43	43.99 ± 28.91	<0.001
25(OH)D (ng/mL)	22.96 ± 7.28	20.96 ± 8.09	<0.001
Femoral neck BMD (g/cm^2^)	0.978 ± 0.098	0.767 ± 0.104	<0.001
LH BMD (g/cm^2^)	1.065 ± 0.106	0.853 ± 0.160	<0.001
LS BMD (g/cm^2^)	1.243 ± 0.146	0.994 ± 0.149	<0.001

FPG, fasting plasma glucose; 2hPG, 2 h plasma glucose; FCP, fasting plasma C-peptide; HbA1c, hemoglobin A1c; SBP, systolic blood pressure; DBP, diastolic blood pressure; TC, total cholesterol; TG, triglyceride; Cr, creatinine; UA, urea acid; BUN, blood urea nitrogen; BMD, bone mineral density; OC, osteocalcin; β-CTX, beta-isomer of the carboxy-terminal cross-linked telopeptide of type I collagen; PINP, procollagen type I N-terminal propeptide; 25(OH)D, 25 hydroxyvitamin D; LH, left hip; LS, lumbar spine.

**Figure 2:**
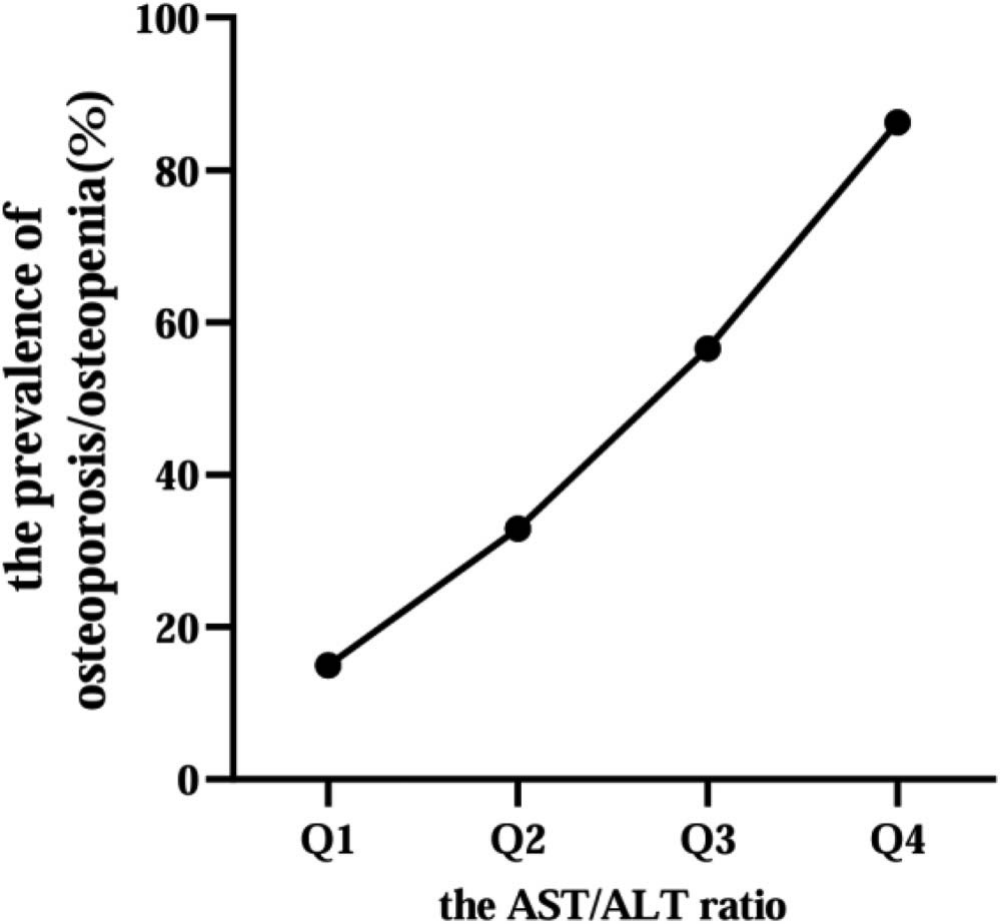
The prevalence of osteopenia/osteoporosis according to AST/ALT ratio quartiles. Q1 14.97%, Q2 32.89%, Q3 56.55% and Q4 86.21%. Trend *P* < 0.01.

**Figure 3:**
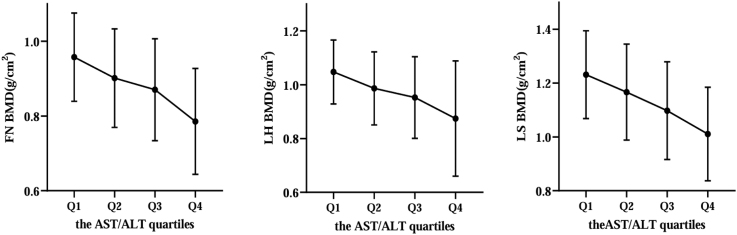
The BMDs differences according to AST/ALT ratio quartiles. FN, femoral neck; LH, left Hip; LS, lumbar spine.

Next, all participants were divided into four groups based on the AST/ALT ratio quartiles, Q1 (0.650, 0.874), Q2 (0.875, 0.999), Q3 (1.000, 1.173) and Q4 (1.174, 1.917). We found that with the increasing rates of the AST/ALT level (Q1 14.97%, Q2 32.89%, Q3 56.55% and Q4 86.21%), the prevalence of osteopenia/osteoporosis increased progressively in T2DM patients (Fig. 2). Meanwhile, the BMDs decreased with the increasing rates of the AST/ALT level (Fig. 3). These trends suggested that the greater the AST/ALT level in T2DM patients, the higher the likelihood of osteopenia/osteoporosis occurrence in those patients. In addition, the results of Spearman’s correlation showed that the AST/ALT levels were significantly negatively associated with femoral neck BMD (*r* = −0.47, *P* < 0.001), LH BMD (*r* = −0.46, *P* < 0.001) and LS BMD (*r* = −0.44, *P* < 0.001) in all subjects (Table 2). However, the correlations between AST/ALT and BMD in Q1∼Q4 groups were different. The AST/ALT level was significantly negatively associated with femoral neck BMD and LH BMD in Q4 group, and was significantly negatively associated with LH BMD in Q1 group. In addition, linear regression analysis also demonstrated the significantly connection of the AST/ALT level to BMDs (Table 3).

**Table 2:** Correlation between AST/ALT level and BMDs in different groups.

AST/ALT	All		Q1		Q2		Q3		Q4	
	*r*	*P*	*r*	*P*	*r*	*P*	*r*	*P*	*r*	*P*
FN BMD	−0.462	<0.001	−0.121	0.143	−0.093	0.257	−0.026	0.755	−0.231	0.005
LH BMD	−0.455	<0.001	−0.169	0.041	−0.080	0.332	−0.037	0.662	−0.190	0.021
LD BMD	−0.445	<0.001	−0.091	0.275	−0.103	0.220	−0.029	0.731	−0.130	0.119

FN, femoral neck; LH, left hip; LS, lumbar spine.

**Table 3:** Linear regression of influence of AST/ALT to BMDs in T2DM.

Variable	Model 1		Model 2		Model 3	
	Beta (95%CI)	*P*-value	Beta (95%CI)	*P*-value	Beta (95%CI)	*P*-value
FN BMD	−0.423 (−0.310∼−0.218)	<0.001	−0.194 (−0.167∼−0.037)	<0.001	−0.189 (−0.162∼−0.074)	<0.001
LH BMD	−0.335 (−0.301∼−0.189)	<0.001	−0.128 (−0.152∼−0.037)	<0.001	−0.335 (−0.147∼−0.036)	<0.001
LS BMD	−0.409 (−0.396∼−0.274)	<0.001	−0.197 (−0.224∼−0.099)	<0.001	−0.409 (−0.196∼−0.079)	<0.001

FN, femoral neck; LH, left hip; LS, lumbar spine.

Model 1 is unadjusted.

Model 2 is adjusted for age, sex, smoking, drinking and BMI.

Model 3 is adjusted for age, sex, smoking, drinking, BMI, SBP, DBP, duration of diabetes, ALT, Cr, UA, OC, β-CTX, PINP and 25(OH)D.

Then, we conducted the logistic regression analysis to further explore the relationship between the AST/ALT level and the risk of osteopenia or osteoporosis. After age, sex, smoking, drinking and BMI adjustment, in comparison to the first quartile of the AST/ALT level (Q1), patients in Q3 and Q4 had a markedly increased risk of osteopenia/osteoporosis (Q3, OR 4.119, 95% CI 2.009–8.085; Q4, OR 17.669, 95% CI 8.272–37.739). After further adjustment for SBP, DBP, duration of diabetes, ALT, Cr, UA, OC, β-CTX, PINP and 25(OH)D compared with Q1 of the AST/ALT level, subjects in Q3 and Q4 remained considerably more likely to have osteopenia/osteoporosis (Q3, OR 3.478, 95% CI 1.641–7.411; Q4, OR 15.278, 95% CI 6.377–36.837) (Table 4).

**Table 4:** Relationship between AST/ALT and osteopenia/osteoporosis risk in different models.

Variable	Model 1		Model 2		Model 3	
	OR (95%CI)	*P*	OR (95%CI)	*P*	OR (95%CI)	*P*
AST/ALT	6.580 (5.561–7.717)	<0.001	6.056 (4.830–7.499)	<0.001	6.257 (4.804–8.405)	<0.001
Subgroups						
Q1	1.00 (reference)		1.00 (reference)		1.00 (reference)	
Q2	3.230 (1.839–5.671)	<0.001	1.854 (0.956–3.594)	0.068	1.500 (0.736–3.054)	0.264
Q3	6.530 (3.733–11.421)	<0.001	4.119 (2.009–8.085)	<0.001	3.478 (1.641–7.411)	0.001
Q4	32.283 (17.008–61.276)	<0.001	17.669 (8.272–37.739)	<0.001	15.278 (6.377–36.837)	<0.001

Model 1 is unadjusted.

Model 2 is adjusted for age, sex, smoking, drinking and BMI.

Model 3 is adjusted for age, sex, smoking, drinking, BMI, SBP, DBP, duration of diabetes, ALT, Cr, UA, OC, β-CTX, PINP and 25(OH)D.

**Figure 4:**
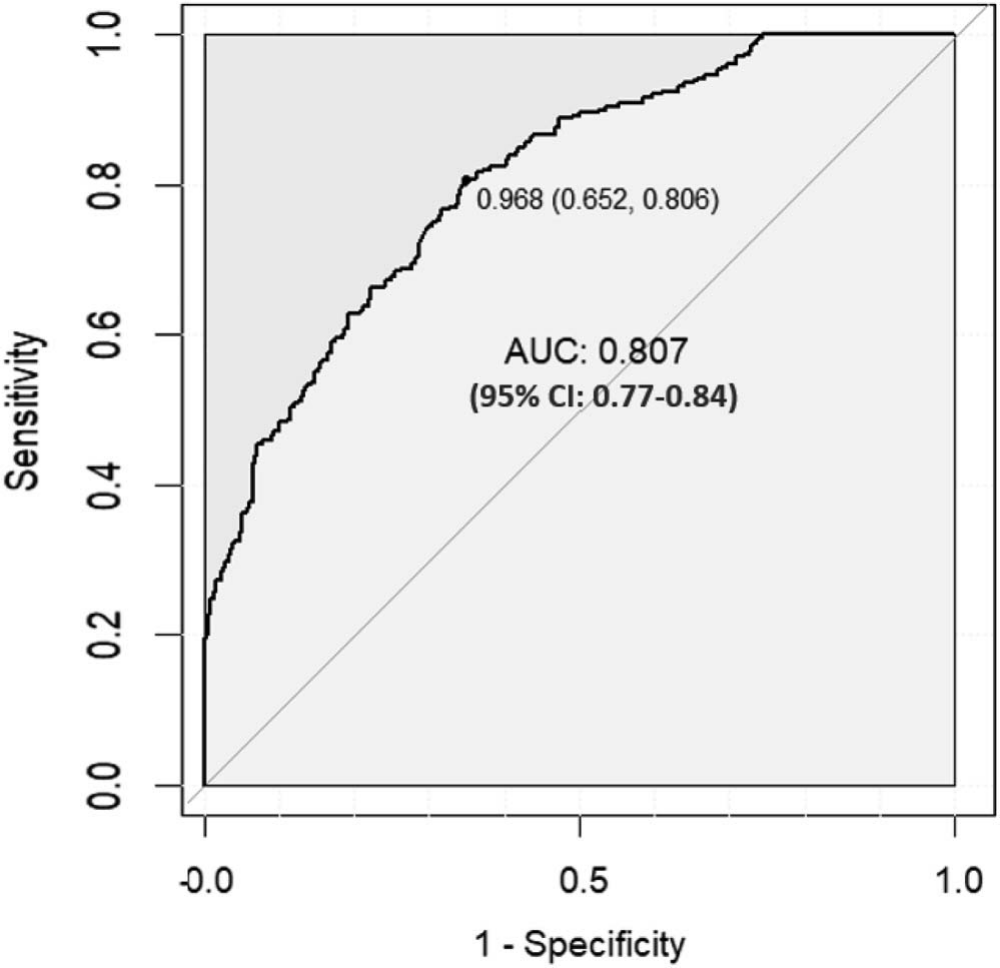
ROC analysis of AST/ALT to predict osteopenia/osteoporosis. AUC = 0.81 (95% CI 0.77–0.84).

The predictive ability of the AST/ALT level about osteopenia/osteoporosis was assessed adopting the ROC curve analysis. The AST/ALT level demonstrated relatively high strength in predicting osteopenia/osteoporosis with AUC = 0.807 (95% CI 0.77–0.84); the optimal cut-off value was 0.968, suggesting that an AST/ALT ratio higher than 0.968 are accompanied by a higher risk of osteopenia/osteoporosis in patients with T2DM (Fig. 4).

## Discussion

In our investigation, we explored the relationship between the AST/ALT ratio and the risk of osteopenia/osteoporosis in patients with T2DM. The present results suggested that the AST/ALT level is negatively associated with BMDs, and the AST/ALT level is associated with an increased risk of osteopenia/osteoporosis in T2DM. When the AST/ALT ratio was analyzed as a variable by quartiles, we observed that the risen prevalence of osteopenia/osteoporosis with the high AST/ALT ratio quartile in the unadjusted model, illustrating the gradient in risk between different quartiles (OR compared with Q1: Q2, OR 3.230, 95% CI: 1.839–5.671; Q3, OR 6.530, 95% CI: 3.733–11.421; Q4, OR 32.383, 95% CI: 17.008–61.276). Whereas, after adjusting for confounders, a strong association with the risk of osteopenia/osteoporosis was demonstrated in Q3 and Q4 groups compared to Q1 (OR 3.478, 15.278). The ROC curve showed that the AST/ALT ratio is a strong predictor of osteopenia/osteoporosis. The results could provide new insights into the complexity of T2DM bone metabolism and contribute to the early detection and risk stratification to improve patients’ bone health.

Patients with T2DM have worse disorders of glucose and lipid metabolism, accompanied by complications such as diabetic micro-/macrovascular disease as the disease progresses (1). These factors have a negative impact on normal bone metabolism, such as disrupting the balance of bone turnover mediated by osteoblasts and osteoclasts, disrupting bone health and leading to a higher risk of osteoporosis and even fractures (3). Osteopenia (decreased bone mass) is the pre-stage of osteoporosis and timely detection and intervention of abnormal bone metabolism is essential. The liver is the metabolic center of the human body, and the occurrence of MAFLD, in addition to affecting the function of the liver, has a close relationship with a variety of extrahepatic diseases that promote each other (7). Previous studies have shown that patients with T2DM are more likely to develop MASLD and have a worse overall metabolic status of the body compared to the healthy population. It is acknowledged that the AST/ALT ratio is an important blood circulation biomarker for assessing liver function. AST has been found to be highly expressed in brain, muscle and kidney tissues, whereas ALT is thought to have higher liver specificity or is abundantly expressed in liver tissues (23). Pathological conditions could lead to abnormal metabolic states in different tissues, resulting in significant abnormal changes in AST or ALT levels, causing the AST/ALT ratio to be an attractive potential marker. Zhai *et al.* reported that MASLD was positively associated with the higher prevalence of osteopenia/osteoporosis and a higher risk of spine fracture (24). Barchetta *et al.* found that obese patients with hepatic fibrosis have lower BMDs, accompanied by degraded bone microarchitecture, suggesting potentially similar pathologic mechanisms between liver and bone in obesity and insulin resistance-related disorders (25). Moreover, evidence suggested that insulin resistance may be the physiopathology mechanism responsible for disturbances in liver function, bone tissue metabolism and skeletal muscle mass and function in patients (26). Numerous studies also have demonstrated that low-grade inflammation and oxidative stress are also contributing factors in the development and progression of MASLD and osteoporosis (11). The liver has a profound impact on bone health and bone tissue also regulates liver function, and these findings imply that it is reasonable to utilize the AST/ALT ratio to aggressively screen and monitor patients with T2DM for osteopenia or osteoporosis.

Our results provided new insights into the relationship between liver enzymes (AST/ALT) and diabetic bone metabolic disorders and a new dimension in understanding possible biomarkers of abnormal bone metabolism in diabetic patients. There are several limitations to this study. First, the sample size of this study was relatively small, and a large cohort study is needed to validate our findings. Second, this study is a cross-sectional study, limiting its causal findings. Specifically, i) lack of temporal sequence: we cannot determine whether the AST/ALT ratio affects bone health first, or whether bone health issues affect the AST/ALT ratio first ii) potential for reverse causality: bone health problems might also influence the AST/ALT ratio, rather than the AST/ALT ratio influencing bone health iii) inability to measure incidence: the cross-sectional study cannot track the sequence of events and thus cannot establish causality, so causality must be assessed through further longitudinal studies and multivariate analysis. Third, the exact biological mechanisms of the relationship between the AST/ALT ratio and osteopenia/osteoporosis remains unclear and requires further study.

## Conclusion

This study demonstrated that the AST/ALT ratio was significantly increased in patients with T2DM combined with osteopenia/osteoporosis, suggesting that the AST/ALT ratio may serve as a useful and reliable biomarker of osteopenia/osteoporosis and emphasizing that more attention should be paid to T2DM patients with the high AST/ALT ratio to further prevent and reduce the occurrence of osteopenia/osteoporosis and related adverse health outcome.

## Declaration of interest

The authors declare that there is no conflict of interest that could be perceived as prejudicing the impartiality of the work reported.

## Funding

This work was supported by the National Natural Science Foundation of China Grant Awards (82374554) and Fundings for Clinical Trials from the Affiliated Drum Tower Hospital, Medical School of Nanjing University (2024-LCYJ-ZXY-02). We would like to thank all participants of the study for their cooperation and support.

## Author contribution statement

Yuan Zhang helped in conceptualization, writing of the original draft, review and editing, formal analysis, methodology, software and visualization. Guanhua Chen and Shanshan Lv helped in writing of the original draft, methodology and formal analysis. Weimin Wang helped with methodology, writing of the review and editing and supervision. Yali Jing helped with writing of the review and editing, conceptualization, resources, supervision, project administration and funding acquisition.

## Ethics declaration

Studies involving human participants were reviewed and approved by the medical research ethics committee of the Nanjing Drum Tower Hospital. Due to the retrospective nature of the study, the medical research ethics committee of the Nanjing Drum Tower Hospital waived the need to obtain informed consent. We confirmed that all experiments were performed in accordance with relevant guidelines and regulations.
